# Understanding the Spatio-Temporal Response of Coral Reef Fish Communities to Natural Disturbances: Insights from Beta-Diversity Decomposition

**DOI:** 10.1371/journal.pone.0138696

**Published:** 2015-09-22

**Authors:** Thomas Lamy, Pierre Legendre, Yannick Chancerelle, Gilles Siu, Joachim Claudet

**Affiliations:** 1 Centre National de la Recherche Scientifique, CRIOBE-USR 3278 CNRS-EPHE-UPVD, 58 Avenue Paul Alduy, 66860, Perpignan cedex, France; 2 Laboratoire d'Excellence CORAIL, 58 Avenue Paul Alduy, 66860, Perpignan cedex, France; 3 Département de sciences biologiques, Université de Montréal, C.P. 6128, Succursale Centre-ville, Montréal, Québec, Canada; 4 Ecole Pratique des Hautes Etudes, CRIOBE-USR 3278 CNRS-EPHE-UPVD, BP 1013, Papetoai, Moorea, French Polynesia; Biodiversity Research Center, Academia Sinica, TAIWAN

## Abstract

Understanding how communities respond to natural disturbances is fundamental to assess the mechanisms of ecosystem resistance and resilience. However, ecosystem responses to natural disturbances are rarely monitored both through space and time, while the factors promoting ecosystem stability act at various temporal and spatial scales. Hence, assessing both the spatial and temporal variations in species composition is important to comprehensively explore the effects of natural disturbances. Here, we suggest a framework to better scrutinize the mechanisms underlying community responses to disturbances through both time and space. Our analytical approach is based on beta diversity decomposition into two components, replacement and biomass difference. We illustrate this approach using a 9-year monitoring of coral reef fish communities off Moorea Island (French Polynesia), which encompassed two severe natural disturbances: a crown-of-thorns starfish outbreak and a hurricane. These disturbances triggered a fast logistic decline in coral cover, which suffered a 90% decrease on all reefs. However, we found that the coral reef fish composition remained largely stable through time and space whereas compensatory changes in biomass among species were responsible for most of the temporal fluctuations, as outlined by the overall high contribution of the replacement component to total beta diversity. This suggests that, despite the severity of the two disturbances, fish communities exhibited high resistance and the ability to reorganize their compositions to maintain the same level of total community biomass as before the disturbances. We further investigated the spatial congruence of this pattern and showed that temporal dynamics involved different species across sites; yet, herbivores controlling the proliferation of algae that compete with coral communities were consistently favored. These results suggest that compensatory changes in biomass among species and spatial heterogeneity in species responses can provide further insurance against natural disturbances in coral reef ecosystems by promoting high levels of key species (herbivores). They can also allow the ecosystem to recover more quickly.

## Introduction

Many ecosystems are subject to recurrent natural disturbances such as fires, hurricanes, outbreaks of predators, diseases or droughts. Characterizing the mechanisms underlying ecosystem response during these major but transient events can be challenging [1]. Natural disturbances can have several contrasting impacts on the populations and communities that are the structural components of these ecosystems. When the disturbance rate does not exceed a given treshold, most communities can maintain the same species composition despite environmental changes [2,3]. However, because the frequency and intensity of natural disturbances are increasing globally [4,5] and because of synergistic effects with additional factors such as anthropogenic stressors [6], productivity [7] and history [8], most communities exhibit changes in species composition as a response to natural disturbances. Hereafter, we will only refer to species composition in terms of quantitative data (e.g. abundance or biomass) as they provide a more complete description of ecosystem and population dynamics.

One of the most common methods to understand community response to natural disturbances consists in assessing the directional changes in species composition along the disturbance gradient—either through time or as a function of environmental changes caused by the disturbance [9]—by computing beta diversity. Yet, changes in species composition between two observations sampled along such a gradient can result from two contrasting mechanisms. If some species decrease (respectively increase) in biomass from one observation to the other, but some other species increase (respectively decrease) in biomass with the same magnitude, replacement among these species will be responsible for the observed change in species composition while the total community biomass will remain the same. Alternatively, if some species decrease (respectively increase) without any reciprocal increase (respectively decrease) in the biomass of other species, then species composition will change solely due to a difference in total community biomass between the two observations. In many studies, the contribution of these two mechanisms are confounded due to the use of a total beta-diversity index, yielding confusing results. However, recent contributions have proposed new ways of decomposing beta-diversity indices computed from quantitative data into two major components, namely a replacement component (also called turnover when analyzed along spatial or environmental gradients) and a biomass difference component [10–13]. First proposed by Baselga [14], the decomposition of beta-diversity has generated an intense methodological debate [10–12] on both the choice of the metrics and the interpretation of each component (e.g. [13,15]). This may have impeded any generalization of the decomposition of beta-diversity indices to resolve important ecological questions. Here, we will focus on the percentage-difference index ([16], section 7.4.2), which is the quantitative equivalent of the Sørensen dissimilarity index, as it reflects the magnitude of changes in species biomass or abundance [9,17]. We will illustrate how this index can be studied through time [18], and also through space [19], and further decomposed into replacement and biomass difference components, the relative importance of which can provide helpful indications on the mechanisms underlying community responses to natural disturbances. While the above-mentioned literature developed and applied beta diversity-decomposition through space, our study is the first one to use it through both space and time and suggest ecological mechanisms that potentially underscore each fraction resulting from the decomposition.

The percentage-difference index can be decomposed to better scrutinize temporal changes in species composition [9,13]. The biomass of all species fluctuates over time due to various processes, including sampling errors, stochasticity, environmental variability or species interactions. The degree to which these fluctuations are correlated among species will determine whether the community is changing due to replacement or difference in total biomass. In particular, if species display high similarity in their response to environmental changes due to the disturbance, they are likely to exhibit correlated fluctuations [20] resulting in a large contribution of the biomass difference component to the total dissimilarity index. However, when populations do not fluctuate in synchrony, the contribution of replacement to total dissimilarity will be larger. Notably, compensatory dynamics between species, whereby species fluctuate non-synchronically over time [21,22], can be an important factor stabilizing community in the face of natural disturbances. Species interactions (e.g., asynchrony in population fluctuations due to competition; [23]) and species-environment interactions (e.g., asynchrony in species environmental responses; [24,25]) can both drive compensatory dynamics. On the one hand, we suggest that assessing the contribution of the replacement component to the percentage-difference index along a disturbance gradient can be useful to assess the importance of stabilizing mechanisms such as compensatory dynamics. This latter mechanism is actually not frequently invoked to capture the ability of natural communities to exhibit stability in the face of environmental changes [1,26]. On the other hand, the biomass difference component quantifies the degree of fluctuation in total community biomass and provides indication on the similarity of species responses to the disturbance.

Further, assessing the spatial variations in community responses is important to comprehensively explore the effects of natural disturbances. Local communities are connected by the dispersal of individuals, which can largely influence local dynamics, reducing [27] or enhancing [28] the temporal fluctuations. Spatial heterogeneity in species responses is an important mechanism, which can provide an insurance effect against natural disturbances [29–31]. However, in habitats impacted by harsh natural disturbances, a large number of species can be filtered out due to their inability to cope with the new environmental conditions without being replaced by other species. As a consequence, when a natural disturbance imposes a strong environmental filtering, spatial homogenization in species composition may consistently occur over time, i.e. species composition becomes similar across local communities [32–34].

Clearly, temporal and spatial responses of communities to natural disturbances can be influenced by various mechanisms but no straightforward method has been suggested to unravel the mechanisms underlying these responses (but see [35]). We suggest that the decomposition of the percentage-difference index into replacement and biomass difference components can be useful in this regard. Though there are no direct mechanistic links between these components and putative mechanisms, the relative contribution of each component can be used as evidence of the importance of stabilizing mechanisms, an example of which is compensatory dynamics. The mechanisms proposed above as possibly responsible for the relative contributions are presented as hypotheses. This is a step forward compared to previous contributions, which simply explained how to compute the decompositions without providing such hypotheses.

As a case study, we used species-rich coral reef communities that experienced two severe natural disturbances. Using data collected from 2004 to 2012 on 13 reefs surrounding Moorea Island (Society Islands Archipelago, French Polynesia), we focused on the response of these communities to the combination of two of the most severe natural disturbances encountered on coral reefs. The first one is an extremely severe crown-of-thorns starfish (COTS; *Acanthaster planci*) outbreak that started in 2006 [36]. COTS is a widespread sea star throughout the Pacific Ocean [37]. It feeds on the living tissues of hard corals [38]. This coral predator proceeds by first colonizing coral reefs inhabited by a large diversity of coral species, frequently referred to as healthy coral reefs. It then produces extremely rapid and intense outbreaks that reduce local coral cover to almost zero within a few years [36,39–42]. Due to its conspicuous impact on the Great Barrier Reef and the associated loss in economic activity, COTS has been classified as serious threat and disaster for coral reef ecosystems [41,42]. In addition, in 2010 a second natural disturbance, a hurricane, impacted the island and decreased the structural complexity still provided by the remaining dead coral [43]. Here, we studied total beta-diversity both in space and time and further decomposed this latter into the relative contributions of replacement and biomass difference components to better scrutinize the response of coral reef fish communities to these two natural disturbances. We first focused on the temporal response at each reef to characterize the relative importance of (1) stability in species composition, (2) compensatory changes in biomass among species and (3) fluctuations in total community biomass. We further investigated the spatial congruence of this pattern by assessing whether compositional changes involved the same species across different reefs.

## Materials and Methods

### Study system

The fish and coral communities of Moorea Island (French Polynesia), located in the South Pacific Ocean, have been studied extensively over the past 30 years [44–50]. In 2004, annual surveys of 13 reefs surrounding the island were initiated [51]. The selected reefs were distributed along the northern (*N* = 4), eastern (*N* = 5) and western (*N* = 4) coasts of the island. Eight of these reefs were designated Marine Protected Area (MPA) in 2004 while the remaining five remained unprotected. However, MPA enforcement only began in 2007 and the ecological effectiveness of this MPA network is still unclear.

### Yearly survey

From 2004 to 2009, each reef was surveyed twice a year along three 50-m^2^ (25×2) random transects on the outer slope at 10 m depth, respectively during the wet (January to March) and dry (July to September) seasons, and once a year from 2010 to 2012 during the wet season. The proportion of live coral encountered beneath 50 equidistant points within each sampling unit was recorded to estimate percent live coral cover. The fish community within each sampling unit was recorded using the fixed count method [52]. In this method, all species seen (including cryptic and juveniles) are counted and sizes are estimated with training errors below 10% of total length. Biomass of each species was computed by applying the length-biomass relationships describes in Kulbicki et al [53]. Because these raw biomass data were heavily skewed, we applied a square-root transformation to reduce asymmetry and compress large biomass values. With this transformation, the analysis of the replacement and biomass difference components is intermediate between analyses conducted on the raw biomass data and on presence-absence data [13].

### Statistical analyses

#### Temporal trajectories of live coral cover, fish biomass and richness

We modeled total biomass (square-root transformed) and species richness as a function of the linear and quadratic effects of time (time and time^2^ using orthogonal polynomials of degree two), space (i.e., reef factor), their interaction (time:reef and time^2^:reef), and season. We used stepwise multiple linear regression models to obtain parsimonious models retaining only the significant effects. To test for the temporal stability in coral and fish communities, we modeled live coral cover, total fish biomass and fish species richness using either a linear, quadratic or logistic effect of time. For each reef, we tested each model and selected the best one to describe the temporal trajectory of coral and fish communities. Indeed, a linear model assumes a constant increase or decrease over time in the response variable at a constant rate estimated by the slope of the effect of time. A quadratic model, on the other hand, makes it possible to model a nonlinear (quadratic) temporal dynamics. Finally, a logistic model assumes that a sudden increase or decrease in the response variable occurred around an inflection point at time *T*; the response variable at time *t*, *Y*_*t*_, was modelled as:

$$Y_{t}=Y_{b}+\frac{Y_{a}-Y_{b}}{1+e^{\frac{T-t}{\sigma }}}$$

where *Y*_*b*_ and *Y*_*a*_ stand for the asymptotic response variable values before and after the inflexion point, respectively. *T* corresponds to the time where inflection occurs, while *σ* is a scale parameter. Model selection was based either on the AIC criterion, or after model comparisons (quadratic *vs* linear models) by ANOVA of the difference between nested models.

#### Temporal trajectory in fish composition

To determine whether temporal changes in fish composition was consistent across reefs, we tested the space-time interaction at the community level using a two-way crossed-factor multivariate ANOVA (by canonical redundancy analysis, RDA) as described in Legendre and Anderson [54] for balanced designs (see also [16], section 11.1.10). Because a significant space-time interaction indicated that temporal evolution was not the same on all reefs, we then used multivariate regression trees (MRT; [55]) to identify the major spatial and temporal groups that exhibited similar fish composition. In addition, to assess whether and when a temporal shift in fish composition did occur on each reef, we also performed chronological clustering by MRT for each reef separately (i.e., time was the only explanatory variable; [56], section 4.11.5). This allowed us to define, for each reef, a pre-shift group and a post-shift group that differed significantly. The proportion of variance explained by that division was measured and tested by RDA.

#### Beta-diversity decomposition

We measured dissimilarity among all pairs of observations using the percentage difference index (also known as the Bray-Curtis index) on square-rooted biomass data. The percentage difference index between observation S_*j*_ and S_*k*_ is defined as:

$$d_{BC}=\frac{B+C}{2A+B+C}$$

where A is the biomass of each species that exists both in S_*j*_ and S_*k*_, B is the biomass of each species unique to S_*j*_ and C the biomass of each species unique to S_*k*_. This dissimilarity index is not Euclidean but the matrix of square-rooted dissimilarities is ([16], Table 7.3).

To test the extent to which each fish composition surveyed in a given reef-time combination was unique compared to the other ones, we computed LCBD indices (i.e. Local Contributions to Beta Diversity). LCBD indices indicate how much each observation has a unique fish composition by assessing its contribution to the total variation in fish composition [17]. To assess the total variation in fish composition, we first computed the total sum of squares (SS_total_) of the matrix of square-rooted dissimilarities by summing the dissimilarities in the lower triangular matrix and dividing by the number of observations *N*. We then computed total beta diversity (BD_Total_) by dividing SS_total_ by *N*–1 [17]. We measured LCBDs directly from the dissimilarity matrix of all observations as the diagonal elements of the matrix containing the centered dissimilarities, divided by SS_total_ [17].

To test whether variations in fish composition were mainly due to compensatory changes in biomass among species or fluctuations in total community biomass, we partitioned total beta diversity into two components, namely biomass replacement (*β*_*replacement*_) and biomass difference components (*β*_*biomassdifference*_). We computed *β*_*replacement*_ following Legendre [13] as:

$$\beta_{replacement}=\frac{2\min\left(B,C\right)}{2A+B+C}$$ (1)

and *β*_*biomassdifference*_ as:

$$\beta_{biomassdifference}=\frac{\left|B-C\right|}{2A+B+C}$$ (2)

Their contributions to total beta diversity were measured by computing the total sums of squares of each matrix (as in the previous paragraph) and dividing them by *N*–1. Beta diversity and its components *β*_replacement_ and *β*_biomasdifference_ were computed for all pairs of sampling units. We repeated the calculations for different stratifications of the data, through time (between temporal steps for each reef) and space (between reefs for each time step). Thus, the sampling units were grouped into 14 temporal groups (with 13 reefs for each time step) and 13 spatial groups (with 14 time steps for each reef).

### Spatial heterogeneity in species responses

Finally, to characterize spatial heterogeneity in the temporal response at the 13 reefs, we computed the indicator value of each species in each of the two temporal clusters defined previously: the pre-shift and post-shift groups [57]. Hence, indicator values indicate which species are predominantly found before or after the disturbances. We adjusted the *P*-values for multiple testing using a sequential Bonferroni correction [58]. Species contributions to temporal shift were then measured as their scores in a RDA testing for the effect of the temporal clusters (represented as a factor in the analysis). Positive scores indicated species that increased in biomass while negative scores identified species that decreased. Hence, the higher the absolute value of a score, the higher the contribution of the species to temporal replacement. Large RDA scores jointly with indicator values were used to select, among the 227 species, a subset of species that were the most representative of the temporal shifts in fish composition occurring on each reef.

All analyses were performed using R 3.0.1 [59]. Nonlinear models were fitted to the data using the *nls* function. MRT were performed using both the mvpart and MVPARTwrap packages. Beta diversity was decomposed into the replacement and biomass difference components using the *beta*.*div*.*comp* function while the LCBD indices were calculated using the *beta*.*div* function (available at http://adn.biol.umontreal.ca/~numericalecology/FonctionsR/). Space-time interaction at the community level was tested using the *anova*.*2way*.*unbalanced* function available from the same source. Indicator values were computed using the labdsv package.

## Results

**Fig 1:**
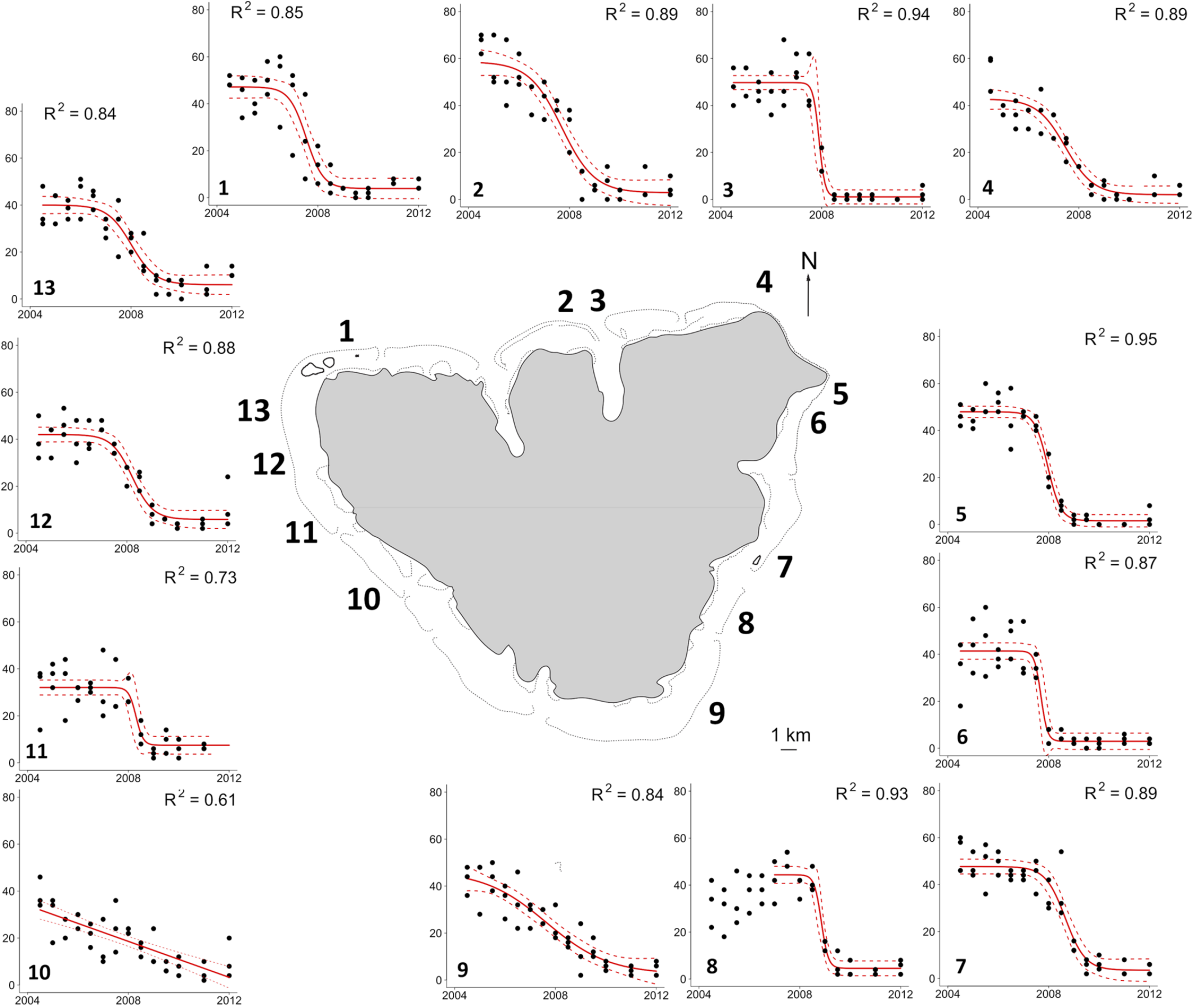
Map of the 13 reefs surveyed from 2004 to 2012 on Moorea Island. Reefs are labelled from 1 to 13 according to their locations. The graphs surrounding the island represent the temporal dynamics in coral cover (in percent) on the corresponding reefs. For each reef, black points represent indiviudal observations; red lines correspond to the fitted logistic models (with the exception of reef 10 for which the temporal dynamic is better represented using a linear model) with their corresponding 95% confidence intervals. *R* ^2^ is the coefficient of determination.

Between 2004 and 2012, the 13 reefs surrounding Moorea Island exhibited large variations in live coral cover (Fig 1 and Supporting Information: S1 Table). Clear dynamics of live coral cover loss over time was evidenced at each reef. All but one reef exhibited a logistic decline in live coral cover over the course of the survey. In 2004, live coral cover was high and stable, ranging from 32% to 59%, but it started to decrease during the first few years of the survey. Between 2007 and 2009 (median = 2008), an inflexion point in the rate of live coral cover loss occurred before it stabilized at very low values ranging from 1% to 7%. Over all reefs, the percentage decrease in live coral cover was 89.7%. Reefs located on the western side (reefs 10–13) of the island experienced slightly reduced loss in live coral cover compared to other reefs around the island (82.8% decline for the western reefs vs. 94.0% for the northeastern reefs; Fig 1 and S1 Table).

Average total fish biomass per reef ranged from 125 g.m^–2^ (95% CI: 98:151 g.m^–2^) to 358 g.m^–2^ (291:425 g.m^–2^; S2 Table and S1 Fig). Mean fish richness ranged from 28.8 species (27.2:30.5 species) to 42.5 species (41.1:44 species). Species richness and total biomass (square-root transformed) were highly variable among observations. Together, time and reef location explained most of that variation (Table 1). Dynamics of fish richness at each reef was well explained using quadratic models (*R* ^2^ ranging from 0.12 to 0.61; S2 Fig), with the exception of reef 9 where the temporal dynamic was better described using a linear model. The species richness on each reef was characterized by a humped curve over time with maximum richness occurring between 2008 and 2010 (i.e., SR_max_ S1 Table). On average, fish richness was maximum 0.75 years after the inflexion point in live coral cover loss (S1 Table).

**Table 1:** Results of multiple regression analyses modeling total fish richness and total fish biomass. Only the significant predictors are shown, along with their partial coefficients of determination (*r^2^*). The time^2^:reef interaction was eliminated from the total biomass model by stepwise selection.

	Total richness				Total biomass			
Predictor	*r^2^*	df	F	*P*	*r^2^*	df	F	*P*
Time	0.07	1	68.6	<0.0001	0.13	1	108.0	<0.0001
Time^2^	0.31	1	209.9	<0.0001	0.05	1	42.5	<0.0001
Reef	0.39	12	27.2	<0.0001	0.13	12	9.3	<0.0001
Time:reef	0.10	12	4.5	<0.0001	0.08	12	5.4	<0.0001
Time^2^:reef	0.05	12	2.2	0.01	-	-	-	-
Season	0.04	1	22.5	<0.0001	0.01	1	11.5	<0.0001
Full model	0.58	39, 506	18.1	<0.0001	0.40	27, 518	12.5	<0.0001

**Fig 2:**
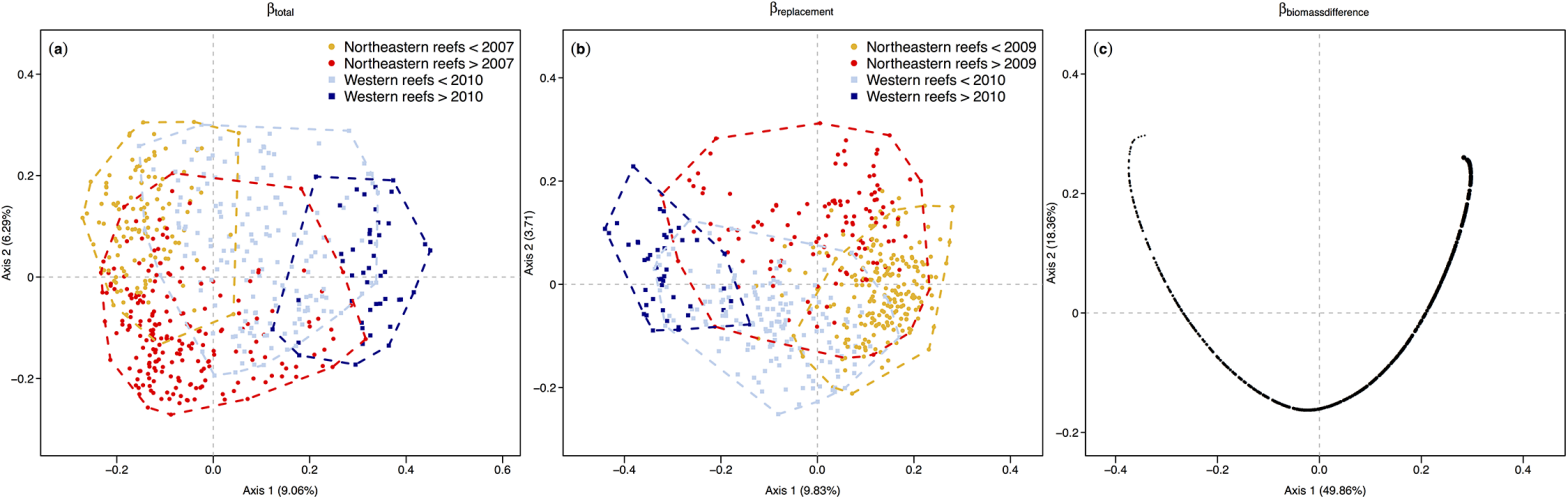
Principal coordinates ordination (PCoA) biplots of beta diversity and its components. (a) PCoA of the square-rooted dissimilarity matrix among all observations. Dissimilarity was measured using the percentage-difference (alias Bray-Curtis) index on square-rooted fish biomass data. (b) PCoA of the dissimilarity matrix accounting only for biomass replacement between pairs of observations (*β*_replacement_). (c) PCoA on the dissimilarity matrix accounting only for biomass differences between pairs of observations (*β*_biomasdifference_). Groups identified in ordinations (a) and (b) are the four groups identified using MRT. In ordination (c), observations are ordered along a total square-rooted biomass gradient (see S3 Fig for details).

Space, time and their interaction significantly explained variation in fish composition based on square-rooted biomass (F_12,364_ = 10.976, *P* < 0.001; F_13,364_ = 6.873, *P* < 0.001; F_156,364_ = 1.593, *P* < 0.001 respectively). Clustering of the observations by multivariate regression tree analysis (MRT) revealed that fish composition was significantly different between reefs on the northeastern coast (reefs 1–9) and reefs on the western coast (reefs 10–13; *R* ^2^ = 6.87% based on RDA). Within each group, a temporal shift occurred in 2007 for reefs located on the northeastern coast (*R* ^2^ = 4.56%) and in 2010 for reefs located on the western coast (*R* ^2^ = 2.71%; Fig 2).

**Fig 3:**
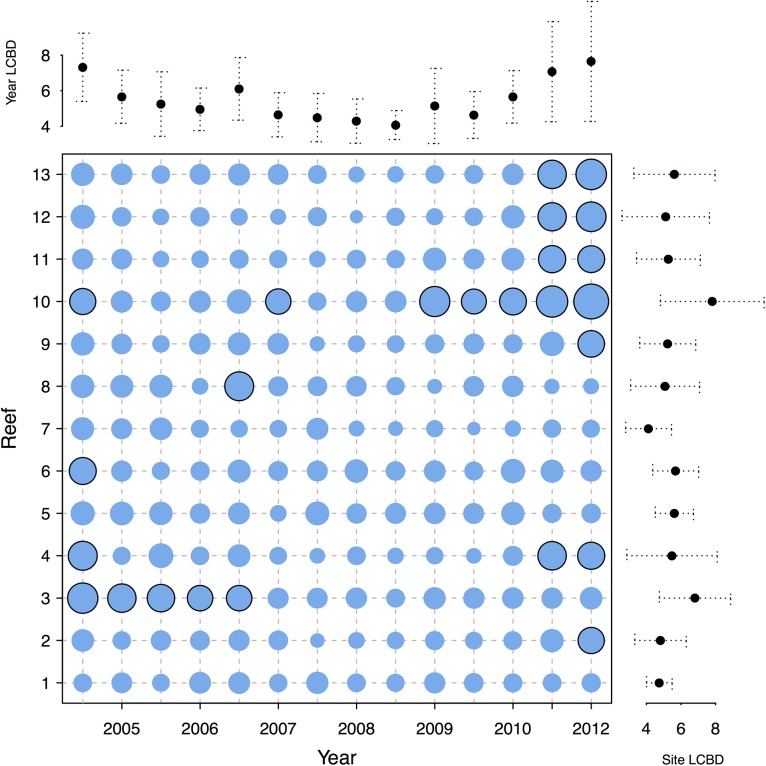
Local contributions to beta diversity (LCBD) per reef and year. LCBD values indicate the extent to which each local community is unique in terms of its composition. Circle surface areas are proportional to the LCBD values. Circles with a black rim indicate significant LCBD indices at the 0.05 level. Marginal diagrams indicate LCBD value averages per year (upper margin) and per reef (right margin); values are multiplied by 1000.

Local contributions of individual observations to beta diversity (LCBDs) exhibited contrasting patterns across space and time (Fig 3). Most of the highest and significant LCBD values were found in the marginal years of the survey, either at the beginning (i.e., 2004 to 2005) or at the end (i.e., 2010 to 2012), away from the inflexion point years shown in Fig 1. For 2011–2012, the reefs located on the western coast (reefs 10 to 13) exhibited higher LCBD values than the other reefs.

A large fraction of fish composition was stable at the scale of the whole island (1 –total beta = 0.698). Changes in fish composition (total beta = 0.302) were mainly due to replacement in biomass among species (*β*_replacement_ = 0.201, accounting for 67% of total beta) whereas fluctuations in total biomass were less important (*β*_biomasdifference_ = 0.101, accounting for 33% of total beta). *β*_replacement_ was explained by the same spatio-temporal variables as total beta diversity: the northeastern reefs were opposed to the western reefs in their replacement (*R* ^2^ = 5.13%) while a temporal shift occurred in 2009 on the northeastern reefs (*R* ^2^ = 2.81%) and in 2010 on the western reefs (*R* ^2^ = 2.01%; Fig 2). *β*_biomasdifference_ was computed as the ratio |B ‒ C| over (2A + B + C) (Eq 4). Because |B ‒ C| is a unidimensional index that orders all observations on a straight line, *β*_biomasdifference_ was exclusively explained by square-rooted total biomass. However, because the denominators (i.e., 2A + B + C) differed among pairs of observations, the resulting ordination was not unidimensional but curvilinear in multivariate ordination space (Fig 2C and S3 Fig). This is clearly illustrated when considering a subset of the data (S3 Fig). For example, observations from reefs 1 and 10 are ordered in the multivariate ordination space by the value of their square-rooted total biomass. In addition, observations from reef 1 mostly have negative coordinates on the first PCoA axis while observations from reef 10 mostly have positive coordinates, reflecting that, overall, reef 1 had much more biomass than reef 10; note that the ordination axis could have been reversed.

**Fig 4:**
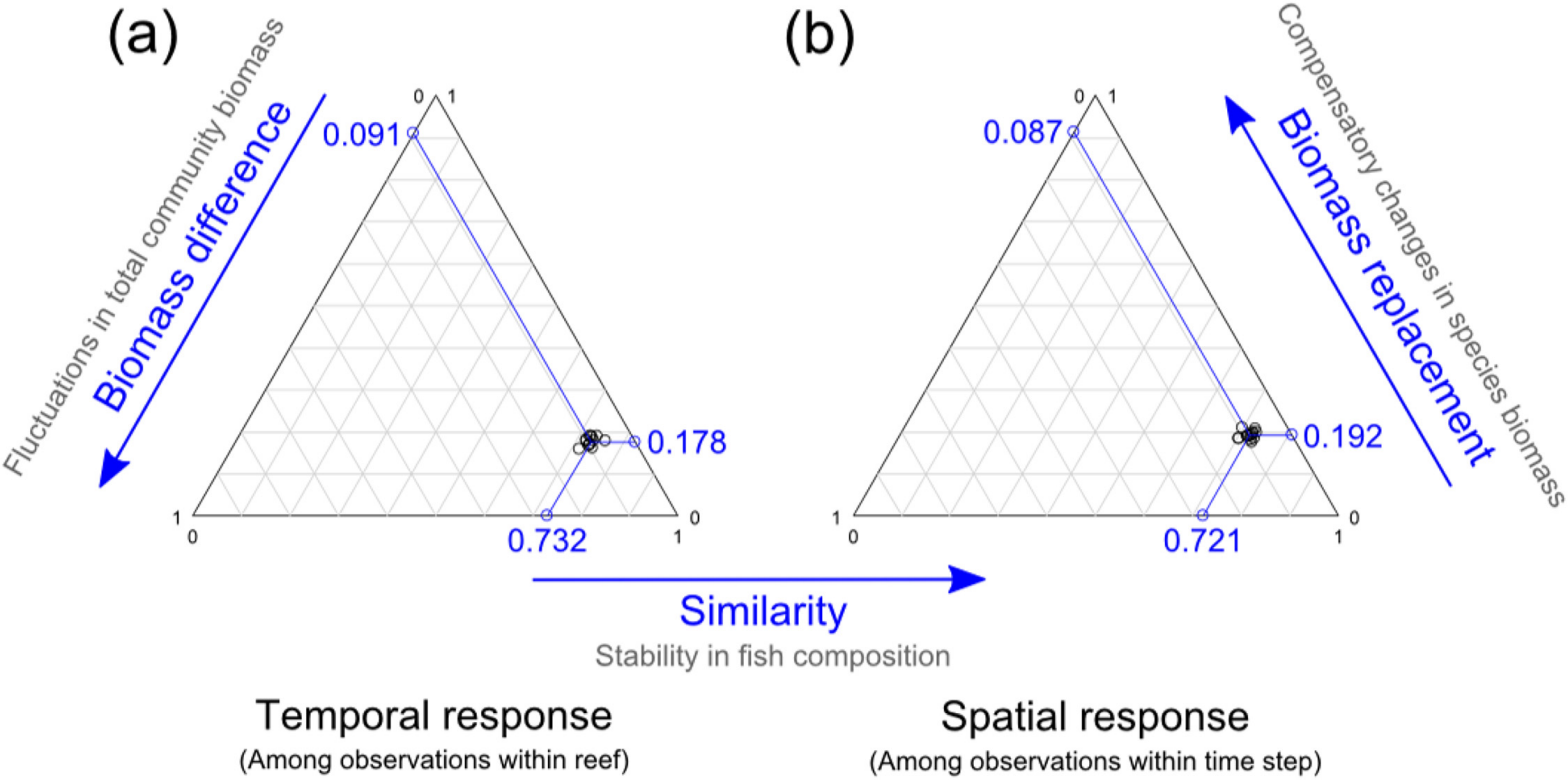
Triangle plots illustrating the contributions of three mechanisms: stability in species composition, compensatory changes in biomass among species and fluctuations in total community biomass to the temporal and spatial responses of fish communities. (a) Temporal response measure as beta diversity for the 13 spatial groups (13 reefs with 14 time steps for each reef). (b) Spatial response measure as beta diversity for the 14 temporal groups (14 time steps with 13 reefs for each time steps). Blue points represent the means over the different time step pairs (or reef pairs).

Using triangular plots, the response of fish communities was then analyzed separately for each of the 13 spatial groups and each of the 14 temporal groups independently. Calculation of beta diversity and its components *β*_replacement_ and *β*_biomasdifference_ within each spatial group provides information on the temporal response at each reef while their calculation within each temporal group provides information on the spatial variation within each time step. Temporal stability in fish composition was highly consistent among reefs (mean similarity across reefs = 0.732, 95% CI = [0.728:0.735]; Fig 4A). Temporal changes in fish composition (beta diversity across reefs = 0.268) were mainly due to biomass replacement among species (*β*_replacement_ = 0.178, 95% CI = [0.175:0.181] accounting for 66% of beta diversity). Spatial stability in fish composition was also highly consistent among time steps and of the same magnitude as the temporal response (average similarity across time steps = 0.721, 95% CI = [0.719:0.725]; Fig 4B). Spatial variation in fish composition (beta diversity across time steps = 0.279) was also mainly due to replacement in biomass among species (0.192, 95% CI = [0.190:0.195] accounting for 70% of beta diversity).

**Fig 5:**
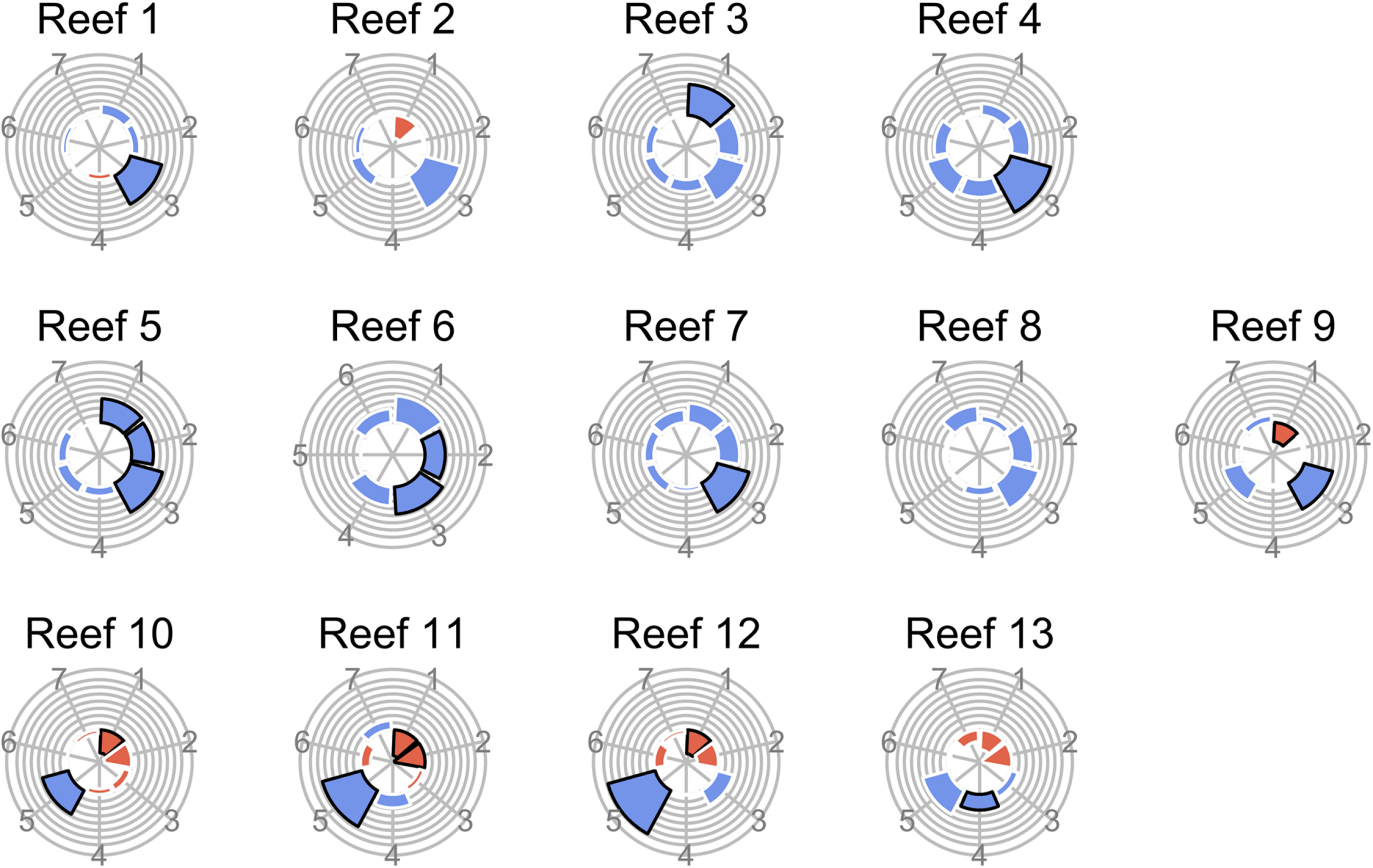
Species contributions to the temporal shift in fish composition for each reef. Only the seven species that contributed the most to this temporal trend are pictured. These species are ordered according to their relative frequencies in the whole data set 1: *Ctenochaetus striatus* (a scraper, Acanthuridae); 2: *Chlorurus sordidus* and 3: *Scarus psittacus* (two scrapers, Scaridae); 4: *Naso lituratus* (a browser, Acanthuridae); 5: *Acanthurus olivaceus* (a grazer, Acanthuridae); 6: *Scarus oviceps* (a scraper, Scaridae) and 7: *Odonus niger* (a plankton feeder, Balistidae). Polygon lengths are proportional to the species contributions to the temporal shift. Positive contributions (in blue) indicate species whose biomasses increased through time, while negative contributions (red) indicate species whose biomasses decreases through time. Polygons of species that are also indicators are surrounded with a black frame (see also S4 Fig).

A temporal shift in fish composition was clearly visible when each reef was analyzed independently (using time as the only explanatory variable: *P* < 0.001 for all reefs; S1 Table). It explained from 8.7% to 19.3% of the variance in fish composition at each reef. We found 35 species out of 227 to be indicators of these shifts (some were significant indicators on several different reefs; S4 Fig). Five of them were extremely rare and were discarded (frequencies < 0.1%). However, overall we found that only a small number of species had a large contribution to the temporal shift. Only seven species explained a large part of the temporal shift, contributing on average to 80% of the total *R* ^2^ explained by the temporal shift on the 13 reefs modeled separately (Fig 5). Among them, the two most prevalent species, namely the Acanthurid *Ctenocheatus striatus* and the Scarid *Chlorurus sordidus*, which represented 9.1% and 6.9% of the total biomass (considering all observations), explained a large part of the temporal shift. Yet, their contribution to the temporal shift exhibited clear spatial heterogeneity (Fig 5). While *Ctenocheatus striatus* and *Chlorurus sordidus* mostly increased in biomass on the northeastern reefs, they did decrease in biomass on the western reefs. In addition, the increase of *Scarus psittacus*, the third most prevalent species (5.2%), explained another large amount of the variation on the northeastern reefs but not on the western reefs. On the latter, *Acanthurus olivaceus* was the species that most contributed to the temporal shift (Fig 5). These four species are all herbivores. On the other hand, few species consistently decreased at the scale of the island (e.g. two highly diet specialized Chaetodontidae: *Chaetodon reticulatus* and *Chaetodon pelewensis*).

## Discussion

All ecosystems inherently experience episodic natural disturbances. For instance, coral reef ecosystems are frequently affected by hurricanes, whereas fires are common in natural forest and grassland ecosystems. Consequently, natural disturbances are key forces shaping the spatio-temporal dynamics of these ecosystems. In addition, as cycles of natural disturbances are already altered due to anthropogenic disturbances [4,5], developing a better understanding of which mechanisms underline the spatio-temporal responses of ecosystems to these disturbances can help ecologists to better inform the management of otherwise degraded ecosystems [60].

The combination of two natural disturbances that severely affected coral habitats around Moorea Island offered a unique opportunity to understand how the coral reef fish communities responded through both time and space. Within a few years, live coral cover decreased logistically by 90% over all 13 monitored reefs. This environmental change occurred at about the same time on the different reefs and corresponded to the start of a bloom of crown-of-thorns starfish (COTS; [36]). This striking impact of COTS on live coral cover outlines the importance of COTS as an important driver of coral dynamics [36,42,49]. Because of the earlier impacts of the COTS outbreak, It is however, more difficult to isolate the part played by the hurricane, which can also cause high coral mortality rates [42,49]. Besides, when COTS feeds on coral tissues, it leaves the mineral coral structure unaltered but corals are then more vulnerable to the mechanical disturbance caused by a hurricane [61]. A recent study in the same area showed that the hurricane decreased the three-dimensional configuration of the reefs [43], potentially reducing the amount of shelters available for benthic fish species.

Our most interesting result revealed that despite major environmental changes, the reef fish communities were extremely resistant to these disturbances. That conclusion emerged consistently whether the response of the coral reef fish community was analyzed globally (among all observations), temporally (among time steps within each reef), or spatially (among reefs within each time step). Stability in reef fish composition underlined an important part of the response (~70%). Temporal variations in coral reef fish composition were mainly explained by replacement in biomass among species (~20%) suggesting that, despite the severity of the two disturbances, fish communities exhibited a high resistance and an ability to reorganize their compositions to maintain the same level of total community biomass as before the disturbances. Different stabilizing mechanisms such as compensatory dynamics may have been at work. As a result, total biomass exhibited little fluctuation over time, which contributed little (~10%) to temporal variation in coral reef fish composition. The compensatory dynamics of local communities were characterized by an increase in biomass of some species and a significant decrease of other species, leading to significant shifts in reef fish composition over time. However, compositional shifts involved changes in different species, especially when comparing the western and northeastern reefs.

Spatial heterogeneity in species responses to the disturbances appears to be an important factor underlying the response of coral reef fish communities to the two disturbances. Compositional shift over time in fish composition was actually mostly ascribed to the consistent increase at the whole-island scale in a group of key species, the herbivores in accordance with a previous study [50]. Although the two disturbances had the same effect on the habitat of all reefs, as characterized by the decline in live coral cover occurring at about the same rate and timing across the different reefs, the identity of the herbivore species fulfilling this critical function differs from site to site. The number of herbivore species identified in this study is quite high (44 species) and may provide the ecosystem with functional redundancy, although herbivory is a complex functional group that includes an increasing number of detailed functions (e.g. grazers, browsers or scrapers). However, because these herbivore species exhibited contrasted temporal trajectories across different reefs despite homogenous environmental change, they probably differed with respect to other traits that influenced their responses. This suggests that there is spatial redundancy in the herbivore response diversity [25,62]. Given the complexity of fish herbivory and the potential role of other functional traits mediating this response, more studies are needed to characterize the functional response of the fish communities. Alternatively, reefs may differ with respect to other environmental characteristics, such as difference in wave exposure, explaining the contrasting temporal trajectories between the western and northeastern reefs. Herbivory plays a critical role in coral reef resilience by controlling algal communities, which may then allow the ecosystem to recover more quickly [63]. This abundance of herbivores probably provided the reef with sufficient herbivory pressure to facilitate coral recruitment and avoid a regime shift towards an algae-dominated system, as observed in other parts of our planet [64,65]. Indeed most of the substrate remained free after the corals disappearance. The extent to which these community changes are naturally occurring in coral reefs, induced by the two disturbances, or intensify due to their joint effects, are however difficult to disentangle. COTS outbreaks and hurricanes have occurred repeatedly at Moorea Island at a decadal scale (e.g. [49]) though this is the first time they occurred in combination over the past few decades.

Finally, despite homogenization of the habitat toward reduced coral cover and complexity, the spatial variation in coral reef fish composition across reefs did not become homogenous over time, as could be the case in some other communities subject to harsh habitat alteration [33,34]. Spatial heterogeneity in species responses may explain why no compositional homogenization occurred. Overall, these spatial responses may provide the ecosystem with greater resilience [66].

Contrary to disturbances associated with human activities such as overfishing, coastal development or extensive land use, natural disturbances are part of the regime under which coral reef ecosystems have evolved [67]. COTS is perceived to be a major threat to coral reefs [41]. Yet, outbreaks of this species are not different from other natural disturbances. COTS has always been a natural predator of corals [68,69] and as such coral reef ecosystems have evolved with this coral predator for a long time. As a natural disturbance, COTS outbreaks also play an important role in the dynamics of these ecosystems [68,70]. However, we have just started to appreciate the influence of COTS at the scale of whole coral reef ecosystems. Recent studies suggested that COTS impact on fish communities can be limited to specialized fish species due either to the loss of their coral habitat (e.g., specialized damselfishes: [71,72] but see [73]) or to negative impact on their recruitment [74]. Our study confirms that the impact of COTS on fish communities can be weak and restricted to some species.

While many studies have documented how species composition varies through space [9,16,75], we have less information about how species composition also varies through time [26,76] though it is a crucial issue given that most ecosystems on Earth are impacted by increasing anthropogenic forces [77]. Undoubtedly, local communities are influenced by processes acting at various spatial (e.g. dispersal) and temporal scales (e.g. competition) but both types of processes are likely to interact so that spatial patterns may vary over time, or alternatively temporal patterns may vary across spatial scales. Monitoring biodiversity both through space and time is then of great interest to obtain a more realistic view of biodiversity changes. Beta diversity analysis is probably the most useful method that can be used to study biodiversity changes through space and time. Although sampling through both time and space is still rare, more time series about various ecosystems are needed to properly document the influence of such episodic natural disturbances on communities. Beta diversity, LCBD indices and beta-diversity decomposition represent useful methods to study spatio-temporal changes.

## Supporting Information

S1 Table Live coral cover (LCC) temporal dynamics and temporal shift in coral reef fish communities. (DOCX)

S2 Table The 13 coral reefs from Moorea Island. (DOCX)

S1 Fig Temporal dynamics in total fish biomass from 2004 to 2012. Black points represent indiviudal observations. Orange lines correspond to the best fitted models, either linears or quadratics, with their corresponding 95% confidence intervals. When no model significantly fitted the temporal evolution of biomass orange dashed lines were used. *R* ^2^ is the coefficient of determination. NS stand for not significant.
(TIFF)

S2 Fig Temporal dynamics in fish richness from 2004 to 2012. Black points represent indiviudal observations. Blue lines correspond to the fitted quadratic models (with the expetion of Maatea for which the temporal dynamic is better represented using a linear model) with their corresponding 95% confidence intervals. *R* ^2^ is the coefficient of determination.
(TIFF)

S3 Fig Influence of the total square-rooted biomass on the ordination of observations when accounting only for biomass differences between pairs of observations. (a) Principal coordinates ordination (PCoA) of the dissimilarity matrix accounting only for biomass differences between pairs of observations (*β*_*biomassdifference*_). For the purpose of illustration we only represented observations from the first transect of reef 1 (green) and 10 (blue). Circles are proportional to the total square-rooted biomass of each observation. 186 and 878 correspond to the minimal and maximal value, respectively, of total square-rooted biomass measured at both reefs. (b) relationship between sampling units coordinates on the first axis (second axis: (c)) of the PCoA and their total square-rooted biomass.
(TIFF)

S4 Fig The 30 indicator species of the temporal shift. Circles are proportional to species score in a RDA with MRT groups corresponding to the temporal shift (i.e., before/after the shift). Black circles indicate significant indicator species. Blue circles correspond to species that increased in biomass after the temporal shift, while red circles correspond to species that decreased in biomass after the temporal shift. Species are classified according to their relative frequencies at the regional (Whole Island) scale (percent within brackets). Species are colored according to their trophic groups: herbivores fish (green); fish feeding predominantly on mobile benthic invertebrates (blue); planktivores (light orange); fish feeding predominantly on sessile invertebrates (red) and piscivores (black).
(TIFF)
