# Supplementary material for: Understanding the Spatio-Temporal Response of Coral Reef Fish Communities to Natural Disturbances: Insights from Beta-Diversity Decomposition
Source: PLoS One. 2015 Sep 22;10(9):e0138696. doi: 10.1371/journal.pone.0138696 (PMC4578945; doi:10.1371/journal.pone.0138696)
Supplement: S1 Table — (DOCX) [file pone.0138696.s001.docx]

**Table S1.** Live coral cover (LCC) temporal dynamics and temporal shift in coral reef fish communities. For each reef, LCC temporal dynamics was modeled as either a logistic or a linear model. For logistic models, *C*_b_ and *C*_a_ are the asymptotic LCC values before and after the inflexion point, respectively. *T* is the estimated year where the inflection occurred, while Δ is the magnitude of LCC loss during the perturbation, measured as the difference between *C*_b_ and *C*_a_. For the linear model, *b* corresponds to the slope, i.e. the amount of coral cover lost per year. *R*² is the adjusted coefficient of determination of each model. Hyphens indicate that the linear model or the logistic model (e.g. reef #10) were not the best fit. In addition, we also provide for each reef the estimated year at which fish richness was maximum (SR_max_) and the estimated year the temporal shift in fish composition occurred (RT). The lag (in years) between the inflection point in coral cover loss (*T*) and the year at which fish richness was maximum is provided in parentheses. Significance of temporal shifts was tested by RDA (*P* < 0.001 for all reefs) while the corresponding adjusted *R*² are shown in brackets in the RT column.

|  | **Coral cover** | | | | | | | |  | **Fish community** | |
| --- | --- | --- | --- | --- | --- | --- | --- | --- | --- | --- | --- |
|  | **Logistic model** | | | | |  | **Linear model** | |  |  |  |
| **Reef** | ***R*²** | ***C*_b_ (95% CI)** | ***C*_a_ (95% CI)** | ***T*** | ***Δ*** |  | ***R*²** | ***b*** |  | **SR_max_** | **RT** |
| 1 | 0.85 | 47.14 (42.36:51.92) | 3.91 (0:8.26) | 2008 | 43.23 |  | - | - |  | 2008.0 (0) | 2009.0 (10.5) |
| 2 | 0.89 | 58.59 (52.46:64.72) | 2.83 (0:8.42) | 2008 | 55.76 |  | - | - |  | 2008.5 (0.5) | 2011.0 (15.9) |
| 3 | 0.94 | 49.75 (46.78:52.72) | 1.07 (0:4.06) | 2008 | 48.68 |  | - | - |  | 2008.0 (0) | 2007.0 (14.1) |
| 4 | 0.89 | 42.76 (38.2:47.32) | 2.02 (0:5.77) | 2007 | 40.74 |  | - | - |  | 2008.5 (1.5) | 2007.5 (19.3) |
| 5 | 0.95 | 47.94 (45.52:50.36) | 1.56 (0:4.18) | 2008 | 46.38 |  | - | - |  | 2009.5 (1.5) | 2009.5 (13.0) |
| 6 | 0.87 | 41.39 (37.91:44.87) | 2.96 (0:6.44) | 2008 | 38.43 |  | - | - |  | 2009.5 (1.5) | 2008.0 (13.2) |
| 7 | 0.89 | 47.70 (44.54:50.86) | 3.56 (0:8.37) | 2009 | 44.14 |  | - | - |  | 2010.0 (1) | 2007.0 (11.1) |
| 8 | 0.93 | 44.44 (40.7:48.01) | 4.55 (1.39:7.71) | 2009 | 39.83 |  | - | - |  | 2009.5 (0.5) | 2007.0 (8.7) |
| 9 | 0.84 | 46.39 (34.65:58.13) | 2.51 (0:10.82) | 2008 | 43.88 |  | - | - |  | 2009.5 (1.5) | 2010.0 (10.7) |
| 10 | - | - | - | - | 28.00 |  | 0.61 | -3.83 (-4.78:-2.88) |  | - | 2008.0 (13.8) |
| 11 | 0.73 | 32.05 (28.87:35.24) | 7.48 (3.68:11.27) | 2008 | 24.57 |  | - | - |  | 2008.5 (0.5) | 2009.0 (16.7) |
| 12 | 0.88 | 42.00 (38.85:45.15) | 5.85 (1.96:9.75) | 2008 | 36.15 |  | - | - |  | 2008.5 (0.5) | 2010.0 (18.6) |
| 13 | 0.84 | 40.03 (36.3:43.76) | 6.08 (1.87:10.3) | 2008 | 33.95 |  | - | - |  | 2008.0 (0.0) | 2010.0 (12.5) |
