# Supplementary material for: Understanding the Spatio-Temporal Response of Coral Reef Fish Communities to Natural Disturbances: Insights from Beta-Diversity Decomposition
Source: PLoS One. 2015 Sep 22;10(9):e0138696. doi: 10.1371/journal.pone.0138696 (PMC4578945; doi:10.1371/journal.pone.0138696)
Supplement: S2 Table — (DOCX) [file pone.0138696.s002.docx]

**Table S2.** The 13 coral reefs from Moorea Island. For each reef, we provide information on its location (latitude and longitude in GWS84) as well as the coast on which the reef is located. We also provide for each reef the mean biomass and richness over years along with their 95% confidence intervals in parentheses.

| **Reef** | **Name** | **Coast** | **Lat** | **Long** | **Biomass (g.m^-2^)** | **Richness** |
| --- | --- | --- | --- | --- | --- | --- |
| 1 | Tiahura | North | -17.483 | -149.900 | 295 (259:331) | 42.5 (41.1:44.0) |
| 2 | Entre 2 Baies | North | -17.476 | -149.837 | 214 (187:241) | 34.9 (33.1:36.7) |
| 3 | Pihaena | North | -17.477 | -149.829 | 257 (217:298) | 32.0 (29.9:34.0) |
| 4 | Aroa | North | -17.471 | -149.777 | 223 (176:270) | 36.1 (33.8:38.4) |
| 5 | Nuarei | East | -17.501 | -149.755 | 358 (291:425) | 41.3 (39.2:43.4) |
| 6 | Temae | East | -17.507 | -149.760 | 291 (229:352) | 40.5 (38.5:42.5) |
| 7 | Ahi | East | -17.549 | -149.771 | 294 (236:351) | 40.7 (38.6:42.7) |
| 8 | Afareaitu | East | -17.569 | -149.784 | 282 (217:347) | 36.6 (34.3:38.9) |
| 9 | Maatea | East | -17.596 | -149.795 | 204 (164:245) | 39.8 (37.6:42.0) |
| 10 | Haapiti | West | -17.554 | -149.898 | 125 (98:151) | 28.8 (27.2:30.5) |
| 11 | Taotaha | West | -17.533 | -149.919 | 258 (218:298) | 34.2 (32.7:35.7) |
| 12 | Tetaiuo | West | -17.512 | -149.926 | 274 (232:316) | 38.2 (36.0:40.3) |
| 13 | Gendron | West | -17.505 | -149.928 | 226 (189:263) | 33.8 (31.9:35.8) |
