# Supplementary figures and images for: Understanding the Spatio-Temporal Response of Coral Reef Fish Communities to Natural Disturbances: Insights from Beta-Diversity Decomposition
Source: PLoS One. 2015 Sep 22;10(9):e0138696. doi: 10.1371/journal.pone.0138696 (PMC4578945; doi:10.1371/journal.pone.0138696)

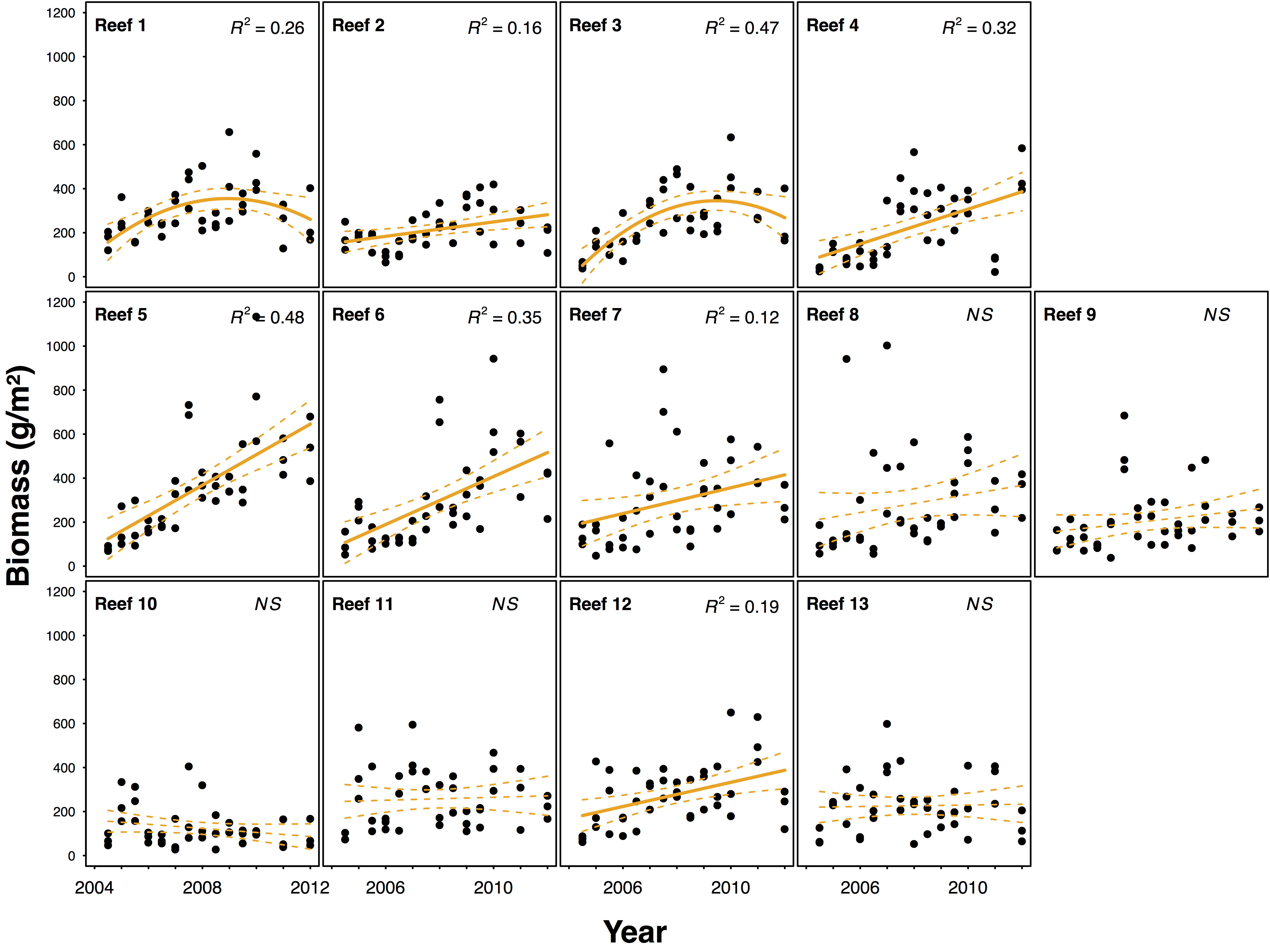

Supplement: S1 Fig — Black points represent indiviudal observations. Orange lines correspond to the best fitted models, either linears or quadratics, with their corresponding 95% confidence intervals. When no model significantly fitted the temporal evolution of biomass orange dashed lines were used. R 2 is the coefficient of determination. NS stand for not significant. (TIFF) [file pone.0138696.s003.tiff]

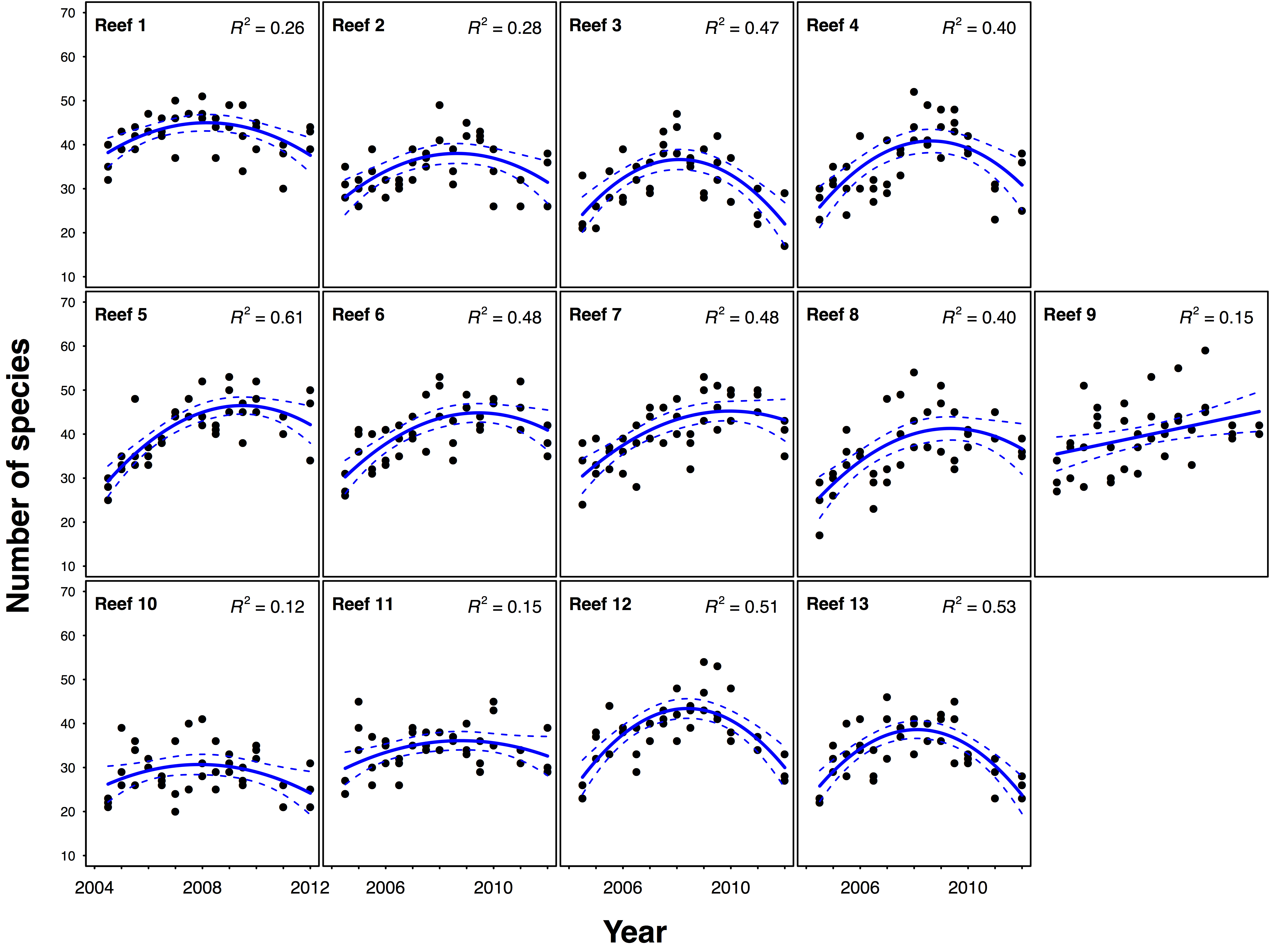

Supplement: S2 Fig — Black points represent indiviudal observations. Blue lines correspond to the fitted quadratic models (with the expetion of Maatea for which the temporal dynamic is better represented using a linear model) with their corresponding 95% confidence intervals. R 2 is the coefficient of determination. (TIFF) [file pone.0138696.s004.tiff]

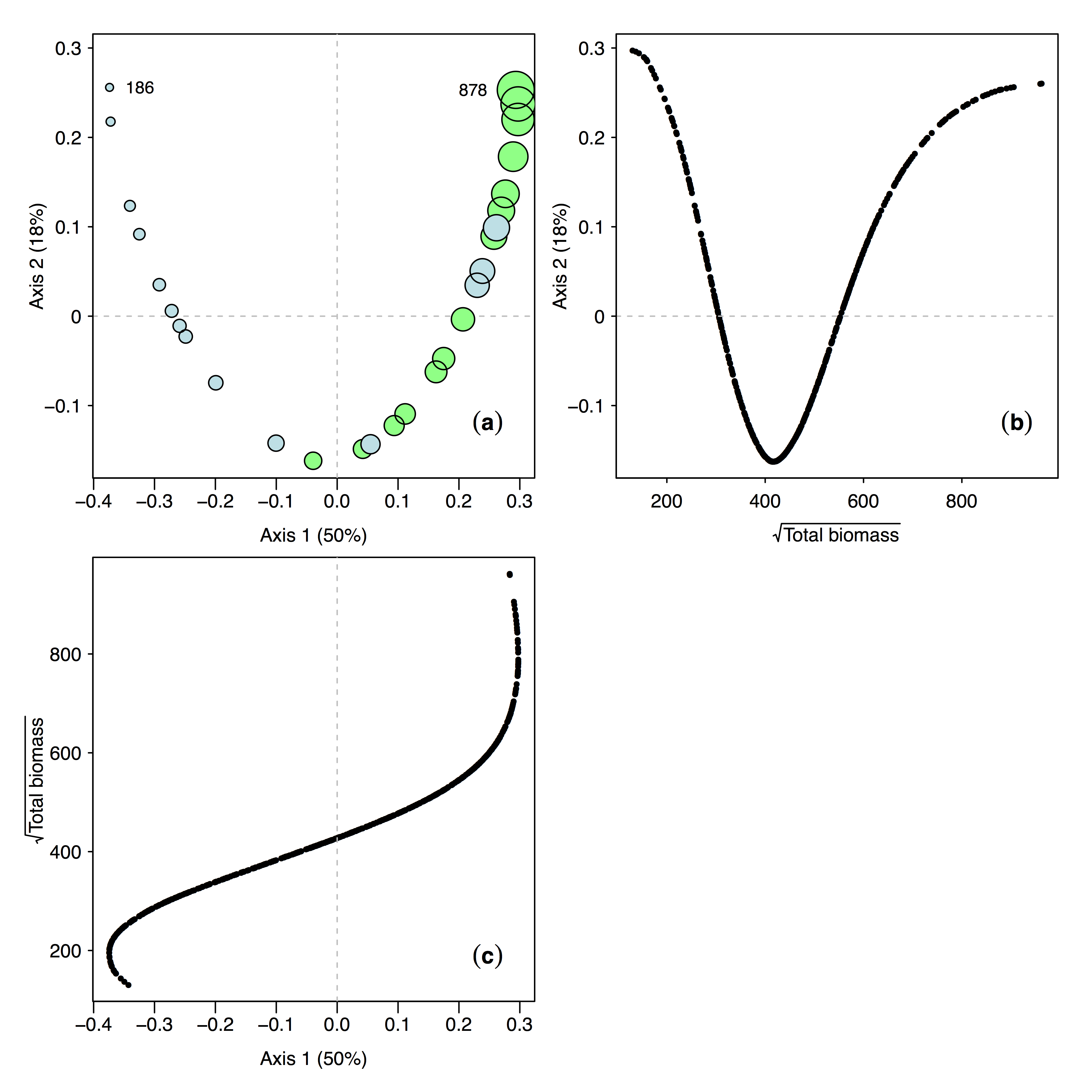

Supplement: S3 Fig — (a) Principal coordinates ordination (PCoA) of the dissimilarity matrix accounting only for biomass differences between pairs of observations (β biomassdifference). For the purpose of illustration we only represented observations from the first transect of reef 1 (green) and 10 (blue). Circles are proportional to the total square-rooted biomass of each observation. 186 and 878 correspond to the minimal and maximal value, respectively, of total square-rooted biomass measured at both reefs. (b) relationship between sampling units coordinates on the first axis (second axis: (c)) of the PCoA and their total square-rooted biomass. (TIFF) [file pone.0138696.s005.tiff]

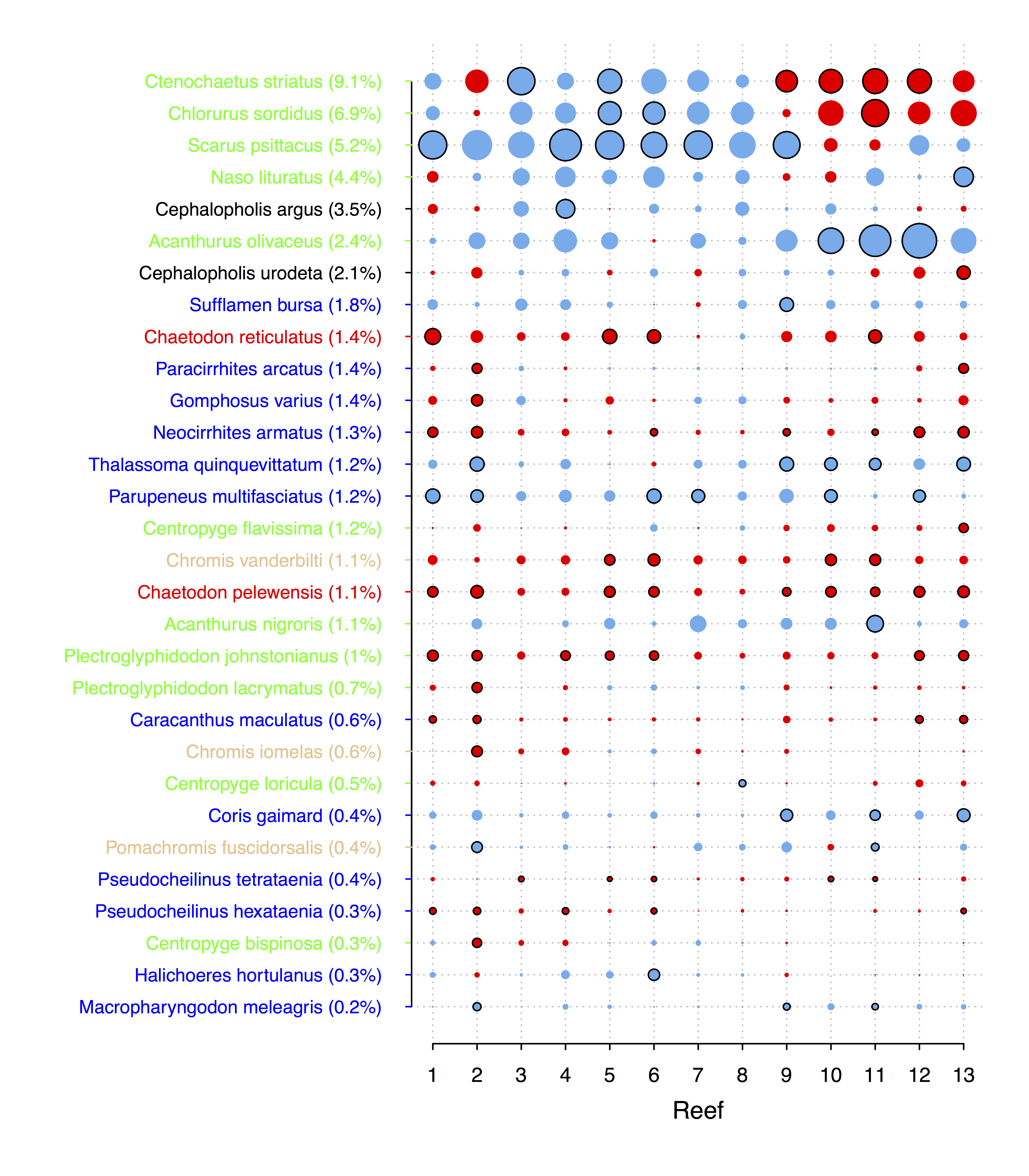

Supplement: S4 Fig — Circles are proportional to species score in a RDA with MRT groups corresponding to the temporal shift (i.e., before/after the shift). Black circles indicate significant indicator species. Blue circles correspond to species that increased in biomass after the temporal shift, while red circles correspond to species that decreased in biomass after the temporal shift. Species are classified according to their relative frequencies at the regional (Whole Island) scale (percent within brackets). Species are colored according to their trophic groups: herbivores fish (green); fish feeding predominantly on mobile benthic invertebrates (blue); planktivores (light orange); fish feeding predominantly on sessile invertebrates (red) and piscivores (black). (TIFF) [file pone.0138696.s006.tiff]
